# Experimental study on axial compression performance of I-shaped aluminum concrete columns embedded in CFRP-PVC square tubes

**DOI:** 10.1371/journal.pone.0330977

**Published:** 2025-09-10

**Authors:** Yuhua Wang, Mengjun Wang, Congrong Tang, Yong Yu, Jiaju Pang

**Affiliations:** 1 School of Electricity and Engineering, Nanjing Vocational Institute of Railway Technology, Nanjing, Jiangsu, China; 2 Jiangsu Xilinghui Construction Engineering Co., Ltd., Nanjing, Jiangsu, China; 3 School of Future Transportation, Guangzhou Maritime University, Guangzhou, Guangdong, China; 4 Guangxi Transportation Investment Group Co., Ltd., Nanning, Guangxi, China; Universiti Teknologi Malaysia, MALAYSIA

## Abstract

To investigate the axial compressive behavior of CFRP-PVC square tube-embedded aluminum concrete columns, five specimens and one control specimen without I-shaped aluminum were tested under uniaxial compression, with the number of CFRP layers and spacing as variable parameters. The failure modes, load-displacement responses, and mechanical properties such as peak load, ductility, stiffness, and energy dissipation were systematically analyzed. Results showed that the incorporation of I-shaped aluminum improved the peak load and ductility by an average of 48.4% and 6.2%, respectively. Increasing the number of CFRP layers increased the bearing capacity by 62.6% for one layer and 69.8% for two layers. Ductility decreased by approximately 15.4% with one layer due to limited confinement but improved by about 15.3% with two layers as the enhanced restraint mitigated stress concentrations and strengthened the composite action. Enlarging the CFRP spacing reduced the bearing capacity by an average of 7.4% but had negligible effect on ductility. The addition of I-shaped aluminum enhanced the axial stiffness by an average of 6.2%, and increasing CFRP layers effectively mitigated stiffness degradation. All specimens exhibited CFRP-PVC or PVC tube rupture and I-shaped aluminum flange buckling, with buckling locations shifting upward as confinement increased. A validated ABAQUS model was used to explore the composite confinement mechanism, and a new confinement model was proposed. The proposed composite columns offer enhanced mechanical performance and durability, making them promising candidates for practical applications in lightweight and high-performance structural elements.

## 1. Introduction

The increasing demand for high-performance structural materials in modern construction engineering, particularly regarding ductility, durability, and seismic resistance, has revealed limitations in traditional concrete and reinforced concrete structures [1]. Concurrently, growing attention to environmental protection and resource reuse has driven the development of sustainable and high-performance building materials. Composite materials, characterized by their exceptional mechanical properties and chemical stability, have thus become a significant focus in structural engineering research [2–4]. Among these, carbon fiber reinforced polymer (CFRP) and polyvinyl chloride (PVC) are notable for their lightweight, corrosion resistance, and superior performance, attracting extensive interest for structural applications [5].

Previous studies have demonstrated that CFRP–PVC tube-confined concrete columns significantly improve compressive strength and lateral–torsional stability, thus enhancing the safety and serviceability of structures [6–9]. Their corrosion resistance and reduced maintenance requirements further support their practical use. However, despite these advantages, such columns often suffer from brittle failure modes and limited ductility, restricting their wider adoption in seismic or dynamic load environments [10–13]. To address these shortcomings, researchers have incorporated internal high-ductility reinforcements. For instance, Xu et al. [14,15] introduced spiral steel reinforcements within CFRP-PVC tubes, demonstrating notable improvements in bearing capacity and ductility through axial compression and seismic performance tests.

Nevertheless, steel reinforcements are susceptible to corrosion, particularly in harsh environments, which compromises long-term durability. Aluminum alloys, with their inherent corrosion resistance and lightweight nature, present an attractive alternative. Prior studies [16–18] have explored the use of I-shaped aluminum sections as internal constraints, effectively eliminating the need for traditional steel reinforcement binding. The composite action between I-shaped aluminum flanges and web plates contributes to enhanced bearing capacity and stiffness of concrete columns. In parallel, extensive research has been conducted on steel-reinforced concrete (SRC) and concrete-encased steel composite columns to predict and enhance their axial performance. Chen and Lin [19] proposed an analytical model for predicting the axial capacity and behavior of concrete-encased steel composite stub columns, while Chen and Wu [20] developed a model for steel-reinforced concrete columns under axial compression. Recent studies by Mostafa et al. [21] and Mostafa [3] have further examined the axial and eccentric behavior of steel-reinforced lightweight aggregate concrete columns, emphasizing the effects of material combinations and column slenderness. These studies provide valuable insights into the interaction between composite materials and concrete, offering a solid theoretical basis for developing new composite column configurations.

Despite these advancements, research on CFRP-PVC tube-confined concrete columns with embedded I-shaped aluminum remains limited. Existing analytical models and experimental findings for steel-reinforced composite columns have not yet been extended to this novel hybrid system, leaving a gap in understanding its confinement mechanism and axial behavior. Therefore, this study proposes a novel composite column configuration combining CFRP-PVC square tubes and I-shaped aluminum reinforcement. Axial compression tests are conducted to investigate the mechanical behavior and feasibility of this composite system, aiming to provide new insights and innovative design strategies for high-performance, durable concrete columns in engineering applications.

## 2. Experimental program

### 2.1 Design of specimen

**Fig 1:**
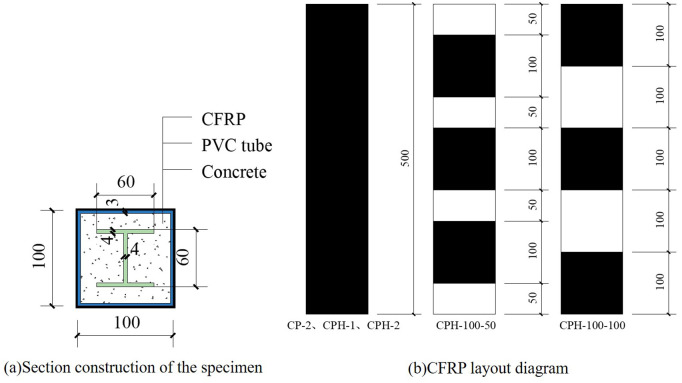
Specimen design.

**Fig 2:**
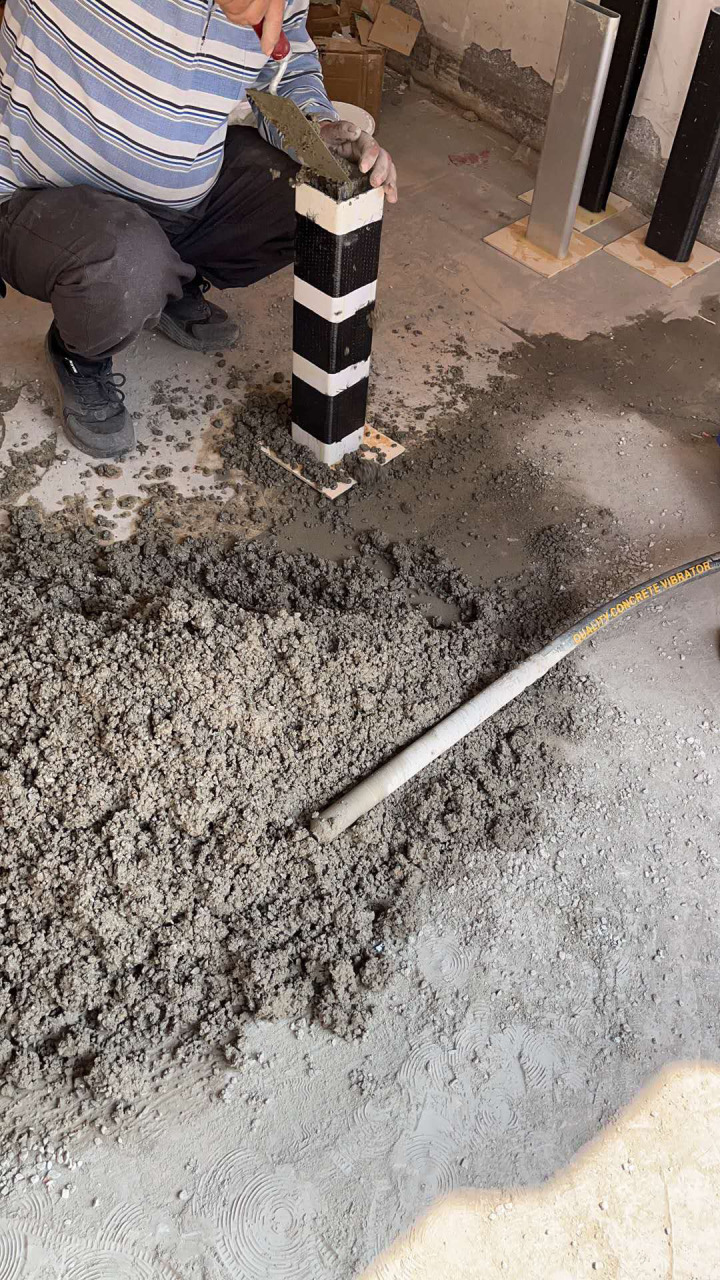
The production process of the specimen.

In this study, five CFRP-PVC square tube-embedded aluminum concrete columns were fabricated, with the primary variables being the number of CFRP layers and CFRP spacing, aimed at investigating the reinforcement effect of I-shaped aluminum. Additionally, one CFRP-PVC tube concrete column specimen without I-shaped aluminum was designed as the control specimen. All specimens have cross-sectional dimensions of 100 × 100 mm and a height of 500 mm. The PVC tubes used in the experiment feature an outer edge length of 100 mm and a thickness of 3 mm. The I-shaped aluminum has a flange width and web height of 60 mm, with a thickness of 4 mm. Fig 1 illustrates the cross-sectional configuration of the specimen, Fig 2 depicts the pouring process, and Table 1 presents the design parameters of the specimens.

**Table 1:** Design of specimens.

Specimen number	The number of layers of CFRP	The width of CFRP strips [mm]	The spacing of CFRP strips [mm]
CP-2	2	500	0
CPH-0	0	500	0
CPH-1	1	500	0
CPH-2	2	500	0
CPH-100–50	1	100	50
CPH-100–100	1	100	100

### 2.2 Raw materials

In this study, C35 grade concrete, compliant with Chinese standard GB 50010−2010 [22], was used. To determine the compressive strength, three standard cube specimens with dimensions of 150 mm × 150 mm × 150 mm were cast and tested, yielding an average compressive strength of *f*_cu_ = 36.8 MPa. The CFRP material, provided by the manufacturer, had a nominal single-layer thickness of 0.167 mm, a width of 100 mm, and an ultimate tensile strength of 3220 MPa. The CFRP and PVC material properties (tensile strength, elastic modulus, etc.) used in this study were obtained from the manufacturer’s data sheets. Although laboratory tests were not performed in this work due to equipment limitations, the adopted values are consistent with those reported in previous experimental studies [23,24]. Future work will include direct testing to further validate the mechanical properties of the CFRP materials employed. For the aluminum components, tensile tests were conducted on three dog-bone specimens in accordance with the Chinese standard GB/T 8804.1−2003 [25]. The average measured yield strength *f*_0.2_ was 196.2 MPa, the ultimate strength *f*_u_ was 212.7 MPa, and the elastic modulus *E*_a_ was 62.15 GPa.

### 2.3 Test equipment and loading system

**Fig 3:**
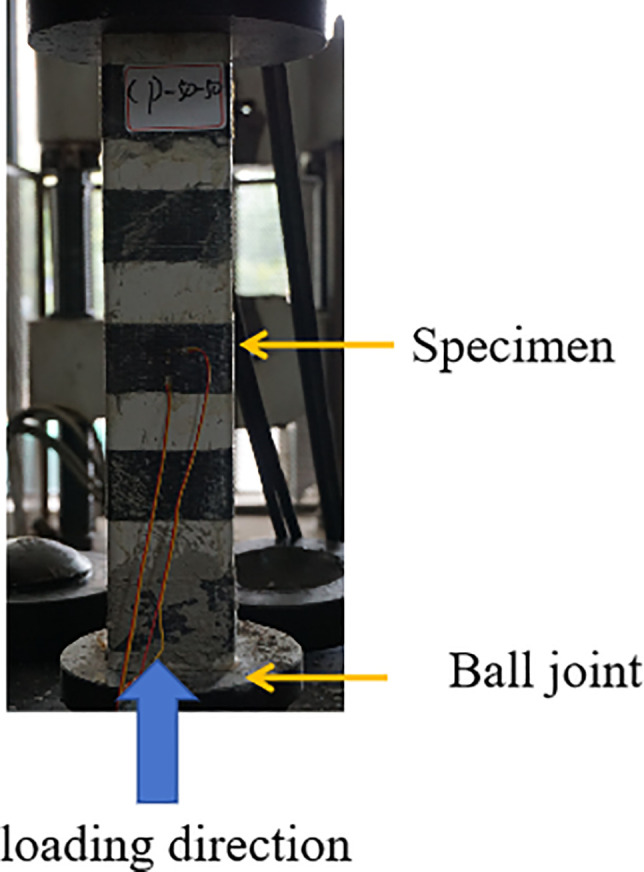
Loading device.

The axial compression test was performed using an electro-hydraulic servo press (designated as YAW-1000), with a maximum axial load capacity of 100 t, as shown in Fig 3. A displacement-controlled loading method was adopted with a loading speed of 2 mm/min, based on the Chinese standard GB/T 50152−2012 [26] for static load testing of building structures. The test was terminated when the load dropped to 70% of the peak value, which corresponds to the post-peak descending stage of the load–displacement curve. Additionally, a secondary termination criterion was set: the test would also stop if the axial displacement reached 5% of the specimen’s height. However, in all tests, the load drop criterion was reached prior to the displacement limit.

## 3. Test results

### 3.1 Failure process and failure mode

**Fig 4:**
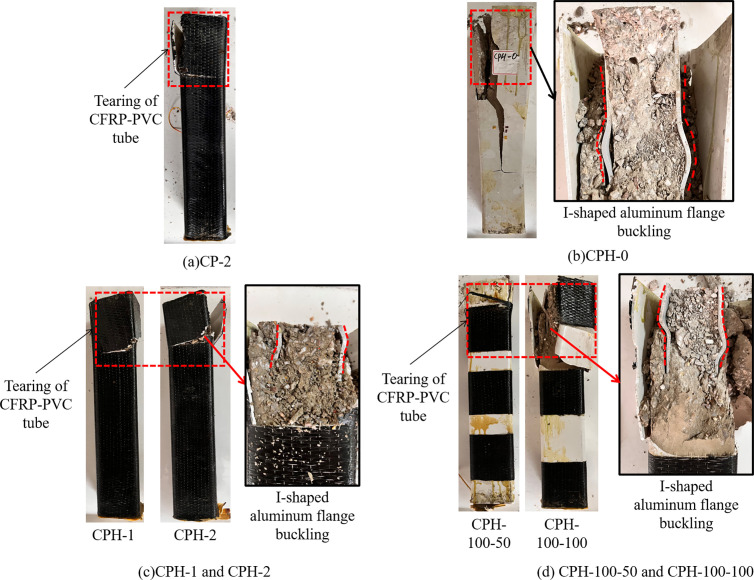
Failure mode.

Fig 4 illustrates the typical failure modes of all specimens, which primarily include external tearing of the CFRP-PVC or PVC tubes and internal buckling of the I-shaped aluminum flanges. In specimen CPH-0 (Fig 4b), which lacked any CFRP confinement, premature and severe local buckling of the I-shaped aluminum flange was observed at mid-height. This failure was attributed to the absence of lateral restraint, causing the flange to lose stability rapidly under axial compression. The flange deformation was pronounced and asymmetric, with significant concrete crushing and spalling along the buckled zone. In contrast, specimen CPH-F (Fig 4c), fully wrapped with CFRP, showed significantly reduced deformation in the aluminum flange, with buckling occurring near the column end. The CFRP-PVC tube remained largely intact until peak load, after which tearing of the CFRP wrapping occurred near the top edge, followed by localized crushing of the confined concrete core. This indicates that continuous CFRP wrapping provides effective confinement and delays local instability. Specimen CPH-S (Fig 4d), which utilized CFRP strips at intervals, exhibited buckling at a location between the mid-height and the top end, representing an intermediate level of confinement. The tearing of the CFRP wrapping was concentrated near the strip ends, where stress concentrations likely initiated premature rupture. Despite partial confinement, the aluminum flanges still showed noticeable lateral deformation, but less severe than that of CPH-0. In the CP-2 control specimen (Fig 4a), which used only PVC tubing without CFRP wrapping, external tearing was also observed. However, the absence of CFRP led to early rupture of the PVC casing and poor confinement of the core, contributing to unstable load-bearing behavior.

**Fig 5:**
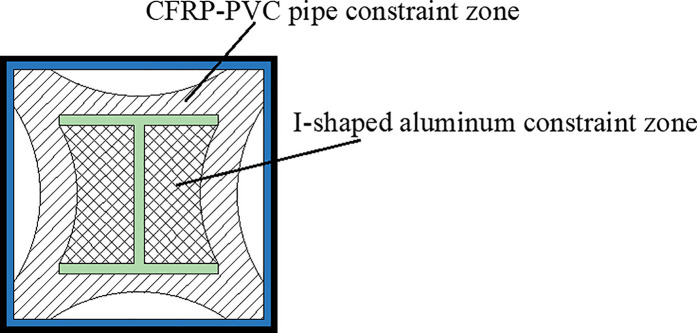
Composite constraint model.

Overall, the failure mode transitions from mid-span buckling to end-localized deformation as the degree of external CFRP confinement increases. These observations suggest that the CFRP wrapping configuration significantly influences the stability and failure characteristics of the embedded aluminum flange. A clear correlation between the confinement level and buckling position can be established: unconfined specimens fail abruptly at mid-height, while well-confined specimens fail more gradually and near the ends. Fig 5 illustrates a conceptual constraint model derived from the observed failure modes and further supported by the finite element analysis presented in Section 4. The outer section of the I-shaped aluminum flange tends to detach after the removal of the CFRP-PVC tube, while the region between the flange and the web remains well bonded, indicating effective concrete confinement in this area. This reinforces the hypothesis that lateral confinement plays a crucial role in preventing flange detachment and enhancing axial load capacity.

### 3.2 Axial load-displacement curve

**Fig 6:**
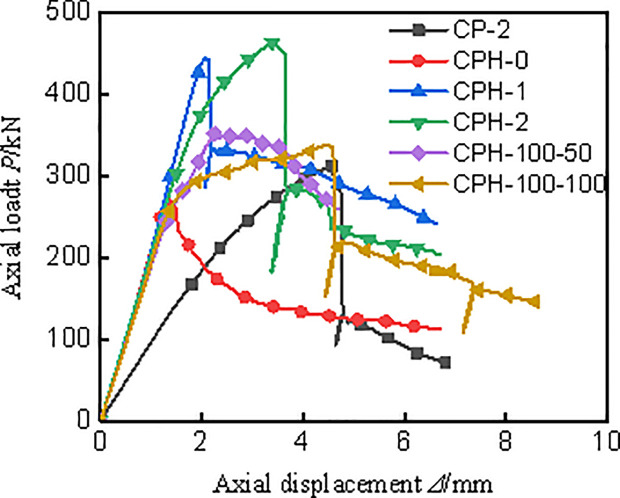
Load-axial displacement curve.

Fig 6 illustrates the axial load-displacement curves for all specimens. As depicted, each specimen progresses through several stages: the elastic stage, the elastic-plastic stage marked by the yielding of the I-shaped aluminum, the steep decline phase due to the tearing of the CFRP-PVC tube, and the gentle phase where the I-shaped aluminum exhibits plasticity. The bearing capacity and axial stiffness of the control group specimen (CP-2) were notably lower than those of the specimens with embedded I-shaped aluminum, underscoring the significant enhancement in axial compression performance provided by the I-shaped aluminum in this composite column. Notably, CPH-1 lacks a distinct elastic-plastic segment compared to CPH-2, displaying more pronounced brittleness. This is attributed to insufficient constraint from the single-layer CFRP, where the I-shaped aluminum has not yet reached its yield strength while the CFRP-PVC tube has already attained its ultimate strength. Therefore, it is advisable to use more than two layers of CFRP. Comparing CPH-2, CPH-100–50, and CPH-100–100, it is observed that as the external constraint capacity diminishes, the slope of the elastic-plastic stage decreases. This occurs because CFRP-PVC tubes and I-shaped aluminum apply three-dimensional compression to the concrete. When external constraints weaken, the confining pressure on the concrete reduces, leading to a decrease in the elastic-plastic slope.

### 3.3 Axial compressive bearing capacity and ductility coefficient

Table 2 summarizes the peak bearing capacity *P*_p_, ductility coefficient *μ*, and axial compression stiffness *K*, all determined from the load-displacement curve. Here, *K* is defined as the secant stiffness at the point corresponding to 0.4*P* on the load-displacement curve [27], while *μ* is calculated using the following method:

**Table 2:** Test results.

Specimen number	*P*_p_ [kN]	*K* [kN ∙ mm^-1^]	*μ*	*E*
CP-2	311.6	96.4	1.27	0.52
CPH-0	272.3	203.7	1.17	0.53
CPH-1	442.9	204.1	0.99	0.57
CPH-2	462.5	205.4	1.35	0.59
CPH-100–50	350.5	189.8	2.02	0.74
CPH-100–100	336.6	188.9	2.04	0.66

*P*_p_ represents the peak axial compressive bearing capacity of the specimen; *K* is the initial axial compression stiffness; *μ* is the axial compression ductility coefficient; *E* is the axial compression energy dissipation factor.


$$\mu =\frac{\Delta_{85\%}}{\Delta_{y}}$$ (1)

**Fig 7:**
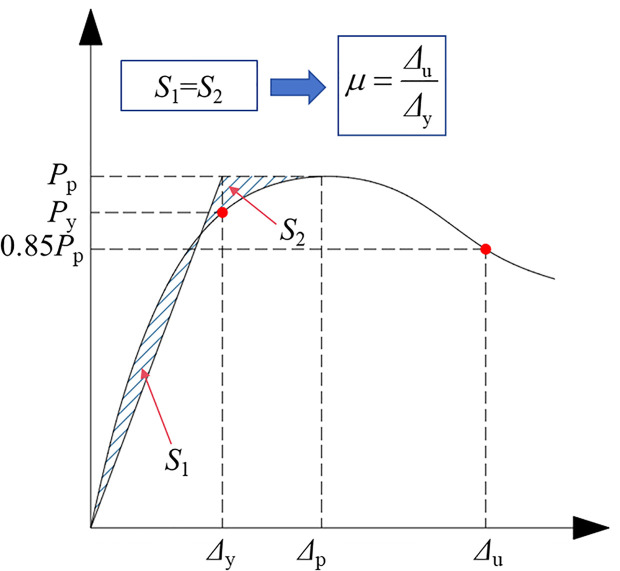
Calculation model of ductility coefficient.

In Eq. 1, the axial displacement (*Δ*_85%_) refers to the displacement when the bearing capacity diminishes to 85% of its maximum value. On the other hand, the yield displacement (*Δ*_y_) can be determined using the equal energy method as described in previous research [28], as shown in **Fig 7**. The yield point can be determined when the area of *S*_1_ is equal to that of *S*_2_.

As indicated in the table, the bearing capacity of the control group specimen (CP-2) is significantly lower than that of the specimen incorporating I-shaped aluminum, demonstrating that the inclusion of I-shaped aluminum substantially enhances the peak load of the composite column. A comparison between CP-2 and CPH-2 shows that the peak load and ductility of CPH-2 increased by 48.4% and 6.2%, respectively. The difference in peak load between the two (150.9 kN) surpasses the theoretical bearing capacity of the I-shaped aluminum alone (134.8 kN), suggesting a synergistic constraint effect between the CFRP-PVC tubes and the I-shaped aluminum on the concrete.

With an increase in the number of CFRP layers, the peak load of the specimen progressively improves. Compared to CPH-0, the ultimate bearing capacities of CPH-1 and CPH-2 increased by 62.6% and 69.8%, respectively, underscoring the effectiveness of CFRP in enhancing the peak load of the composite column. However, the rate of improvement diminishes when transitioning from 1 to 2 layers of CFRP. The axial compressive ductility of the specimen initially decreases and then increases with the addition of CFRP layers. This trend may arise because, with fewer CFRP layers, the restraining effect on the PVC tubes and I-shaped aluminum-concrete columns is limited. In such instances, the axial compression ductility is primarily governed by the properties of the concrete and PVC tubes, with CFRP providing minimal reinforcement. This limited reinforcement can lead to stress concentrations between the PVC tubes and concrete, potentially reducing overall ductility. When the number of CFRP layers increases to 2, the restraining effect becomes more pronounced, effectively distributing external loads, mitigating stress concentrations, and enhancing the synergistic interaction between the PVC tubes and concrete columns. At this stage, the increased number of CFRP layers improves structural ductility, achieving optimal reinforcement and significantly enhancing axial compression ductility.

Compared to CPH-100–50, increasing the CFRP spacing in CPH-100–100 results in a 4% decrease in peak load due to weakened external constraints. However, ductility increases by 0.9%, with minimal overall change. This is because the ductility of the specimen is primarily provided by the I-shaped aluminum. Thus, under weaker external constraints and unclear changes, the ductility contribution from the I-shaped aluminum remains dominant.

### 3.4 Stiffness degradation

**Fig 8:**
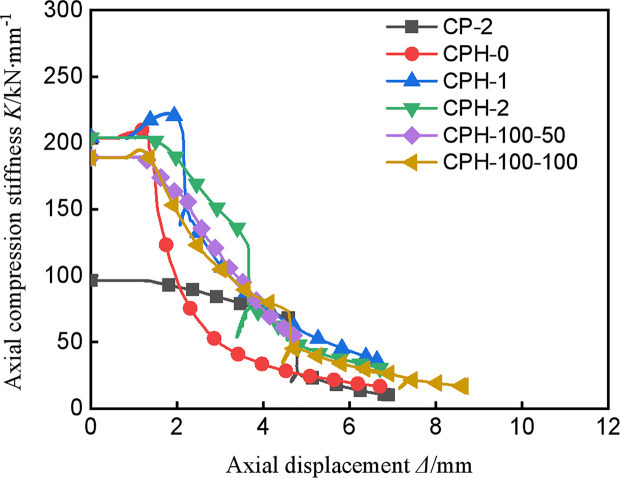
Stiffness degradation curve.

**Fig 8** presents the stiffness degradation curves for all specimens, and **Table 2** lists the calculated initial axial compression stiffness K values. As illustrated in **Fig 8**, the stiffness degradation curves of all specimens exhibit a similar trend: a rapid decline when the axial displacement is less than 4 mm, followed by a tendency to flatten. This behavior is primarily attributed to the plasticity of the I-shaped aluminum. Notably, CPH-0 exhibits the fastest rate of axial compression stiffness degradation, mainly because, in the absence of CFRP constraints, the damage progression in concrete is accelerated, leading to a quicker stiffness reduction.

**Table 2** details the initial axial compression stiffness for each specimen. A comparison between CP-2 and CPH-2 reveals that the axial compression stiffness of CPH-2, which includes built-in I-shaped aluminum, increased by 113.1%, indicating that the presence of I-shaped aluminum significantly enhances the axial compression stiffness of the composite column. As the number of CFRP layers increased from 0 to 2, the stiffness degradation of the specimens was mitigated, and the initial axial compression stiffness gradually increased, albeit with a maximum increase of only 0.6%. This is because, under axial compression load, the axial compression stiffness of the specimen is predominantly provided by the concrete and I-shaped aluminum, with external constraints having a relatively minor influence on stiffness. Comparing CPH-100–50 and CPH-100–100, it was observed that the axial stiffness curves of the specimens largely overlapped, and the initial stiffness values were also nearly identical. This further underscores that external constraints have a relatively small impact on axial stiffness.

### 3.5 Energy dissipation factor

**Fig 9:**
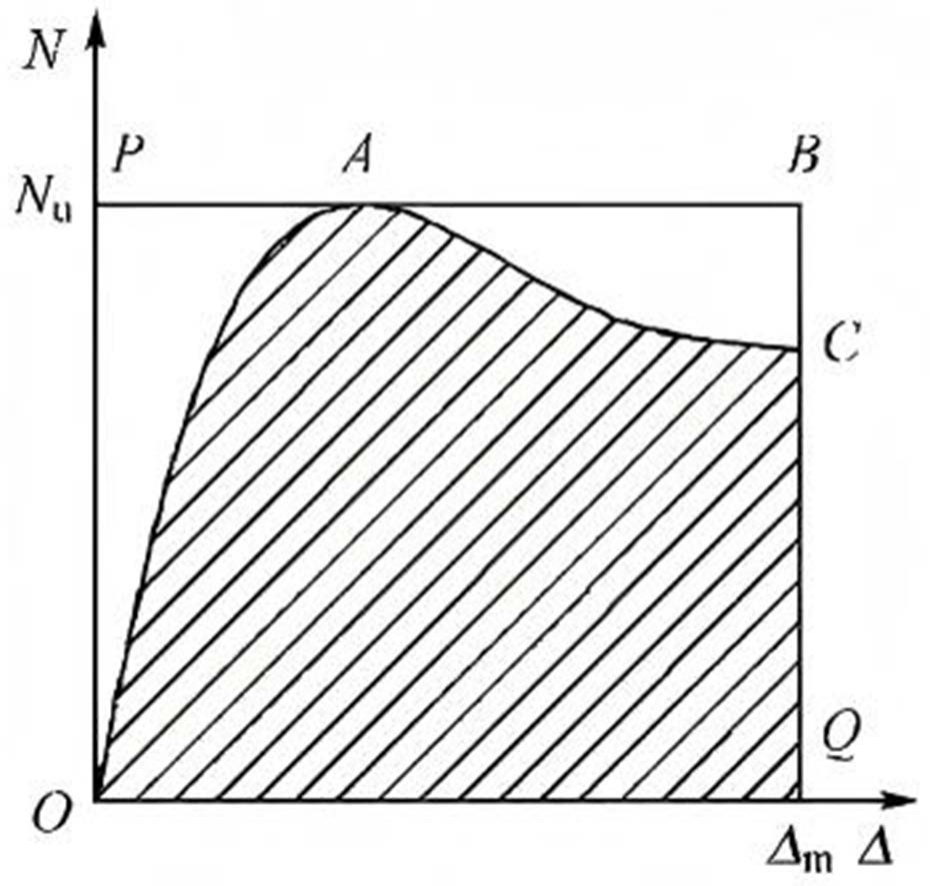
Energy dissipation factor.

The energy dissipation capacity reflects the inherent relationship between energy absorption and energy dissipation of axial compression components themselves, and has global variable significance [29]. The axial compression energy dissipation factor *E* of the specimen is defined as:


$$E=\frac{S_{OACQ}}{S_{OPBQ}}$$ (2)

where *S*_OPBQ_ is the area enclosed by the axial load displacement curve parallel to the horizontal axis and the vertical coordinate axis passing through the termination point. *S*_OACQ_ is the external rectangular area as shown in **Fig 9**. *N*_u_ is the peak load. *Δ*_m_ is the displacement at the end of the experiment.

The calculation results of energy dissipation factors for all specimens are shown in **Table 2**. Compared with CP-2 and CPH-2, the energy dissipation factor of CPH-2 with built-in I-shaped aluminum has increased by 13.4%. This is because I-shaped aluminum profiles themselves have high strength and stiffness, and being embedded in concrete columns can effectively disperse and transmit axial loads, allowing the columns to absorb and dissipate more energy during compression. On the other hand, I-shaped aluminum profiles can form a skeleton structure inside concrete columns, improving the stress distribution inside the concrete. This optimized stress distribution can reduce stress concentration, delay local structural failure, and thus improve the energy dissipation capacity of the entire column. Meanwhile, CFRP-PVC tubes can form external constraints on concrete columns, limiting the lateral deformation of concrete. Under the action of external constraints, the overall structure can more effectively absorb and dissipate energy.

With an increase in the number of CFRP layers, the energy dissipation factor of the specimen exhibits a rising trend. Relative to CPH-0, the energy dissipation factors of CPH-1 and CPH-2 have risen by 7.5% and 113%, respectively. Decreasing the spacing of CFRP also enhances the specimen’s energy dissipation capacity. When the spacing is reduced from 100 mm to 50 mm, the energy dissipation factor increases by 12.1%. This improvement is attributed to the greater number of CFRP layers and narrower spacing, which strengthen the external constraint on the specimen. This enhanced constraint more effectively restricts the lateral deformation of both the concrete and the I-shaped aluminum profiles, improves the overall structural stability, prevents stress concentration, and allows the structure to absorb and dissipate more energy.

## 4. Finite element analysis

Although the number of specimens was limited due to cost and fabrication complexity, the selected configurations effectively represented the key influencing parameters, and the findings were further validated through numerical simulations. In this study, the ABAQUS finite element software was employed for modeling and analysis. ABAQUS is capable of performing comprehensive simulations of concrete structures, including nonlinear material behavior, complex interactions, and damage evolution, thereby providing reliable and detailed insights that complement the experimental results [30,31].

### 4.1 Constitutive properties of materials

#### 4.1.1 CFRP.

In this study, Hashin Damage [32] was used to describe the fracture damage behavior of CFRP, and the calculation method is as follows:


$$F_{f}^{t}={\left(\frac{\sigma_{11}}{X^{T}}\right)}^{2}+\alpha {\left(\frac{\tau_{21}}{S^{L}}\right)}^{2}-1\leq 0,\sigma_{11}\geq 0$$ (3)

$$F_{f}^{c}={\left(\frac{\sigma_{11}}{X^{C}}\right)}^{2}-1\leq 0,\sigma_{11}\leq 0$$ (4)

$$F_{m}^{t}={\left(\frac{\sigma_{22}}{Y^{T}}\right)}^{2}+{\left(\frac{\tau_{12}}{S^{L}}\right)}^{2}-1\leq 0,\sigma_{22}\geq 0$$ (5)

$$F_{m}^{c}={\left(\frac{\sigma_{22}}{2S^{L}}\right)}^{2}+\left[{\left(\frac{Y^{C}}{2S^{L}}\right)}^{2}-1\right]\frac{\sigma_{22}}{Y^{C}}+{\left(\frac{\tau_{12}}{S^{L}}\right)}^{2}-1\leq 0,\sigma_{22}\leq 0$$ (6)

where σ_11_, σ_22_, τ_12_ were the stress component; *X*^T^, *X*^C^ were the tensile strength and compressive strength along the fiber length; *Y*^T^, *Y*^C^ were the tensile strength and compressive strength of the fiber in the vertical direction; *S*^L^ was the shear strength. The meanings and calculation methods of the above symbols can be found in Reference [15], the Hashin damage model parameters from **Table 3** were employed.

**Table 3:** CFRP material properties.

Elastic parameters						Hashin’s damage Model [MPa]					
*E*_1_	*E*_2_	*υ*_12_	*G*_12_	*G*_13_	*G*_23_	*X* ^*T*^	*X* ^*C*^	*Y* ^*T*^	*Y* ^*C*^	*S*_*12*_	*S*_*13*_
211.49	7.93	0.35	5.3	5.3	4.0	3300	1414	134	169	134	120

*E*_1_ and *E*_2_ are the elastic modulus [GPa], *υ*_12_ is the Poisson’s ratio, *G*_12_, *G*_13_ and *G*_23_ are the shear modulus [GPa], respectively.

#### 4.1.2 Aluminum alloy.

In this study, the stress-strain relationship of aluminum alloy proposed by Ramberg Osgood [33] was adopted, these parameters can be found in Wang et al [34]. The calculation method is as follows:


$$\varepsilon =\left\{ \begin{array}{c} \frac{f}{E_{a}}+0.002{\left(\frac{f}{f_{0.2}}\right)}^{n}\;\;\;\;\;\;\;f\leq f_{0.2}\\ \frac{\left(f-f_{0.2}\right)}{E_{0.2}}+{\left\{\frac{0.008-\left(f_{1.0}-f_{0.2}\right)}{E_{0.2}\frac{\left(f-f_{0.2}\right)}{\left(f_{1.0}-f_{0.2}\right)}}\right\}}^{n\prime_{0.2,1.0}}\;f\geq f_{0.2} \end{array}\right.$$ (7)

In Eq. 7, in addition to the symbols mentioned in section 2.2, *f* and *ε* represent the stress and strain of aluminum alloy, respectively; *E*_0.2_ is the secant stiffness corresponding to *f*_0.2_.

#### 4.1.3 Concrete.

In this study, the concrete damage plasticity (CDP) model was adopted. In the CDP model, the material parameters used for concrete analysis include the elastic modulus (*E*_c_) and Poisson’s ratio (ν). The Poisson’s ratio was assumed to be 0.2 and remained constant for both uncracked and cracked concrete. The influence of different compressive and tensile yield stresses on the deviatoric yield surface was considered through the shape parameter (*K*_c_), which was set to 0.667 to satisfy the convexity condition (0.5 ≤ *K*_c_ ≤ 1) [34]. The plastic flow was defined using the dilation angle (*ψ*) and the eccentricity parameter (*∊*), where the plastic volumetric strain generated during plastic shearing was controlled by *ψ*. The dilation angle was assumed to remain constant during plastic yielding and was observed to range between 25° and 45°; in this model, *ψ* was set to 32° [35,36]. The eccentricity parameter *∊* allowed for an increase in *ψ* under low confining pressure in uniaxial loading conditions and was set to 0.1 in this study. For concrete, the ratio of biaxial to uniaxial compressive strength (*σ*_b0_/*σ*_c0_) was taken as the default value of 1.16 to account for the material behavior under multiaxial stress conditions [37]. In ABAQUS, it is necessary to define the compressive and tensile behavior of concrete. The constitutive relationship of concrete under compression adopts the full curve equation of uniaxial compression specified in GB50010−2010 [18]:


$$y=\left\{ \begin{array}{c} ax+(3-2a)x^{2}+(a-2)x^{3}\;0\leq x\leq 1\\ \frac{x}{b{(x-1)}^{2}+x}\;\;\;\;\;\;\;\;\;\;x\geq 1 \end{array}\right.$$ (8)

$$y=\frac{\sigma }{f_{c}},x=\frac{\varepsilon }{\varepsilon_{c}}$$ (9)

$$\varepsilon_{c}=\left(700+172\sqrt{f_{c}}\right)\times 10^{-6}$$ (10)

In the Eq. 8, *f*_c_ and *ε*_c_ are the axial compressive strength and peak strain of concrete, respectively.

The tensile constitutive curve of concrete is calculated using the following formula:


$$y=\left\{ \begin{array}{c} 1.2-0\mathrm{.2}x^{6}\;0\leq x\leq 1\\ \frac{x}{c{(x-1)}^{1.7}+x}\;\;x\geq 1 \end{array}\right.$$ (11)

$$y=\frac{\sigma }{f_{t}},x=\frac{\varepsilon }{\varepsilon_{t}}$$ (12)

$$\varepsilon_{t}=65{f_{c}}^{0.54}\times 10^{-6}$$ (13)

In the Eq. 11, *f*_t_ and *ε*_t_ are the tensile strength and peak tensile strain of concrete, respectively.

#### 4.1.4 PVC.

The stress-strain response and softening properties of PVC plastic differ significantly from those of concrete. PVC plastic demonstrates notable ductility and can endure substantial deformation. As an isotropic material, it exhibits uniform behavior in all directions. In the model, the PVC tube was simulated as a von Mises material incorporating isotropic hardening. The elastic modulus, yield strength, and ultimate strength were determined based on material tests performed in this study.

### 4.2 Mesh

**Fig 10:**
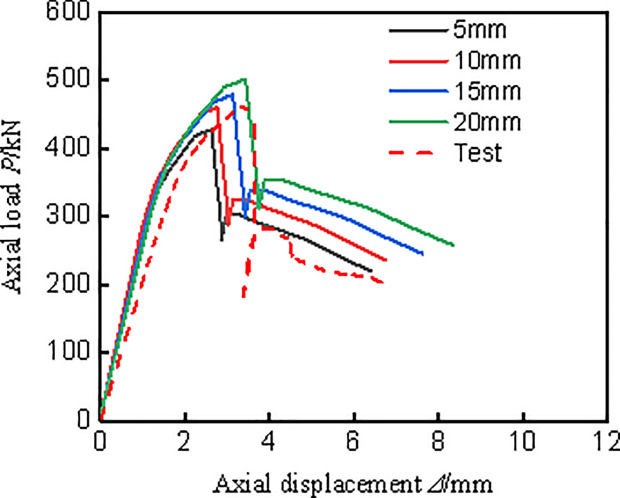
Comparison of simulation results for different mesh sizes.

Concrete and PVC tubes use C3D8R elements, while CFRP and I-shaped aluminum employ S4R elements. A mesh sensitivity analysis was carried out using element sizes of 5 mm, 10 mm, 15 mm, and 20 mm, as shown in **Fig 10**. The results showed that while the 5 mm mesh provided slightly higher accuracy, the difference in key output parameters (e.g., peak load and failure mode) between the 5 mm and 10 mm meshes was minimal (less than 3%). On the other hand, larger mesh sizes (15 mm and 20 mm) led to noticeable discrepancies and less precise damage localization, particularly in the CFRP layers. Therefore, a 10 mm mesh size was adopted globally to ensure a good balance between computational efficiency and accuracy. This mesh size is also sufficient to capture the fracture behavior of the CFRP sheets effectively.

### 4.3 Interaction and boundary conditions

**Fig 11:**
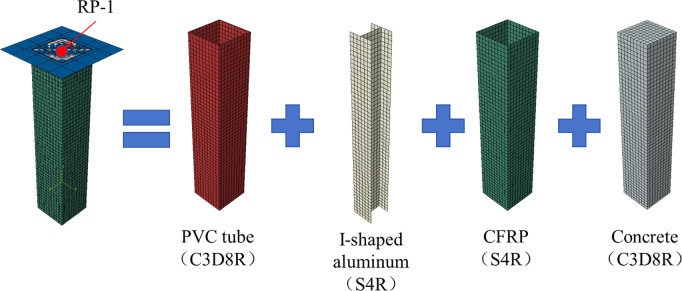
Modeling of specimen.

During the experiment, no bond failure was observed between the CFRP and the PVC tube, leading to their interaction being defined as a tie. The interface between the PVC tube and concrete is typically characterized as face-to-face contact, with hard contact in the normal direction and a penalty function governing tangential behavior, incorporating a friction coefficient of 0.25 [15]. To replicate the experimental loading conditions, the lower section of the specimen was constrained as a fixed support, while the upper section was assigned a hinged boundary condition. Specifically, the hinge was modeled by restraining the out-of-plane displacements and axial rotations at the top end of the specimen, allowing only vertical displacement to simulate the application of axial loading. This setup ensures consistency with the spherical hinge used in the physical tests and enables accurate replication of boundary conditions in the numerical model. Axial displacement was applied via a reference point on the loading plate, which was coupled to the top surface of the specimen to ensure uniform load transfer. A Static, General step with nonlinear geometry enabled was used to simulate the axial compression process. The second-order effects were accounted for by modeling one end of the specimen as hinged, allowing rotations to develop during loading. The finalized model is illustrated in **Fig 11**, where the location and coupling of the reference point are clearly shown.

To account for potential geometric imperfections, an initial out-of-straightness with an amplitude of *H*/1000 (where *H* is the specimen length) was introduced in the finite element model. This value is commonly adopted in column analyses. However, due to the relatively short length of the specimens used in this study, the introduction of this imperfection had an insignificant influence on the overall load–displacement response and failure mode.

### 4.4 Model validation

**Fig 12:**
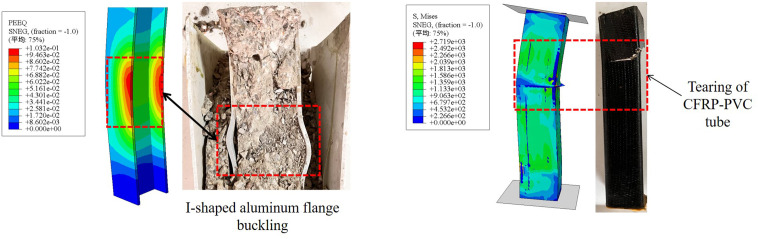
Comparison of failure modes.

The failure modes of two typical specimens CPH-0 and CPH-2 are shown in **Fig 12**. The finite element simulation results demonstrate a high degree of consistency with the experimental observations. Stress concentration and tearing occur at the corners of the CFRP-PVC tubes, while significant plastic deformation is observed at the flange of the internal I-shaped aluminum.

**Fig 13:**
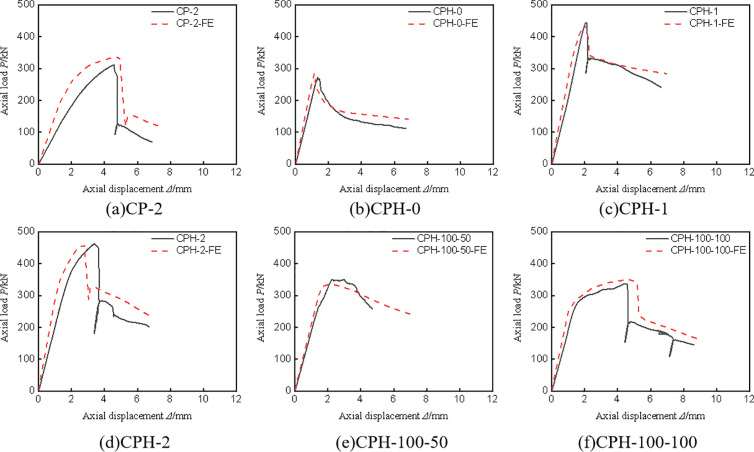
Comparison between test measurement curves and finite element calculation curves.

**Fig 13** presents a comparison between the load-displacement curves obtained from finite element simulations and experimental measurements. The simulation results exhibit high accuracy, with the curve trends and peak loads closely aligning with the experimental data.

Additionally, **Table 4** summarizes the peak axial compression bearing capacities derived from finite element calculations, showing an average error of 1.4% and a maximum error within 9.6%. These results indicate that the developed finite element model is highly reliable and can be utilized for further mechanism analysis.

**Table 4:** Comparison between finite element results and experimental results.

Specimen number	*P*_p_ [kN]	*P*_FE_ [kN]	*Δ*_p_ [mm]	*Δ*_p,FE_ [mm]	*P*_FE_/ *P*_p_	*Δ*_p,FE_/ *Δ*_p_
CP-2	311.6	336.2	4.58	4.91	1.079	1.072
CPH-0	272.3	283.5	1.33	1.83	1.041	1.376
CPH-1	442.9	400.2	2.03	2.11	0.904	1.039
CPH-2	462.5	452.9	3.40	2.77	0.979	0.815
CPH-100–50	350.5	337.6	2.22	1.64	0.963	0.739
CPH-100–100	336.6	320.7	4.40	4.63	0.953	1.052
Average					0.986	1.016
Standard deviation					0.063	0.224

### 4.5 Constraint mechanism analysis

**Fig 14:**
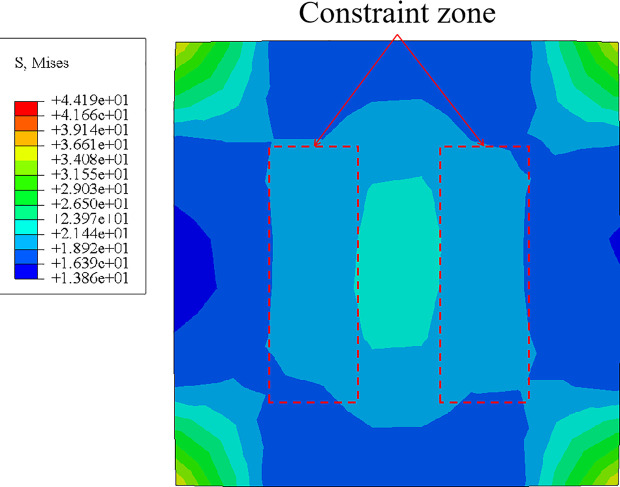
Section stress distribution.

To further investigate the constraint mechanism of this composite column, the stress distribution in the mid-span cross-section of the CPH-2 specimen was analyzed, as illustrated in **Fig 14**. The stress distribution indicates that the four sides of the section experience relatively low stress, whereas the corner regions exhibit significantly higher stress levels. This suggests that the constraint effect is weaker along the four sides but stronger at the corners, resembling the constraint characteristics of square steel tube concrete columns [38–42]. However, a notable distinction is the significant increase in stress in the region where the I-shaped aluminum is located, particularly in the constraint zone between the flange and web of the I-shaped aluminum. This observation indicates that the CFRP-PVC tube provides a composite constraint in conjunction with the I-shaped aluminum, preventing concrete detachment in this region. Based on these constraint characteristics, a composite constraint model is proposed, as shown in **Fig 5**.

### 4.6 Parameter analysis

**Fig 15:**
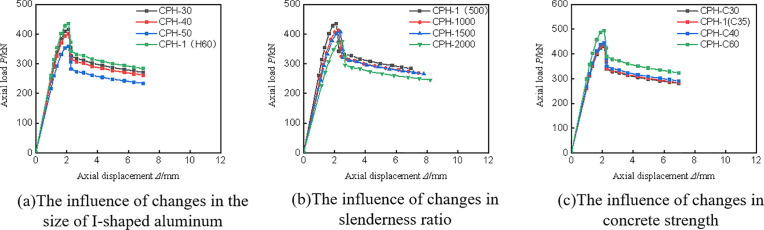
Load displacement curve of parameter analysis specimen.

To address the limitations of the experiment, ABAQUS finite element software was utilized to save both time and cost. In this study, the CPH-1 specimen was selected as the reference model, based on which the effects of varying I-shaped aluminum dimensions, specimen heights, and concrete strengths on the peak load and failure mode of composite columns were analyzed. The design parameters and calculation results for all specimens are presented in **Table 5**, the load displacement curve is shown in **Fig 15**.

**Table 5:** Parameter analysis of specimen design parameters and calculation results.

Specimen number.	*I*-shaped aluminum size [mm]	*H* [mm]	Slenderness ratio *H/B*	Concrete strength [MPa]	*P*_FE_ [kN]	Note
CPH-1	60 × 60 × 4	500	5	36	400.2	Test specimen
CPH-30	30 × 30 × 4	500	5	36	339.3	Changes in dimensions of I-shaped aluminum
CPH-40	40 × 40 × 4	500	5	36	354.6	
CPH-50	50 × 50 × 4	500	5	36	366.0	
CPH-1000	60 × 60 × 4	1000	10	36	379.9	Change in aspect ratio
CPH-1500	60 × 60 × 4	1500	15	36	375.4	
CPH-2000	60 × 60 × 4	2000	20	36	346.4	
CPH-C30	60 × 60 × 4	500	5	30	396.0	Changes in concrete strength grade
CPH-C40	60 × 60 × 4	500	5	40	409.7	
CPH-C60	60 × 60 × 4	500	5	60	476.1	

**Fig 16:**
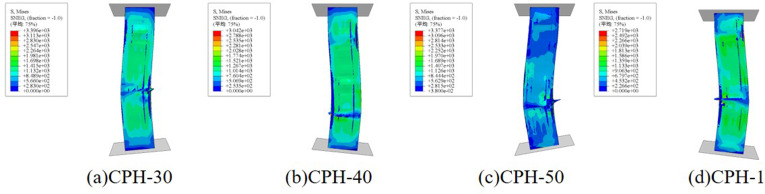
The influence of changes in the size of I-shaped aluminum on the failure mode.

**Fig 16** illustrates the influence of different I-shaped aluminum dimensions on the failure mode of composite columns. The results indicate that variations in aluminum size have minimal impact on failure modes, with CFRP-PVC tube tearing predominantly occurring in the middle and lower regions of the specimen. However, as the size of the I-shaped aluminum increases, the peak load of the column improves correspondingly. Specifically, compared to the smallest section (H30), the axial compressive bearing capacity increased by approximately 4.5% for H40, 7.8% for H50, and 17.8% for the largest H50 section used, demonstrating a significant enhancement in load capacity with increasing aluminum cross-sectional dimensions.

**Fig 17:**
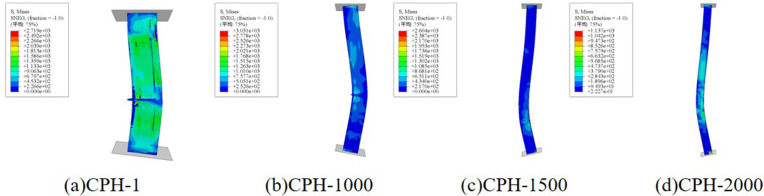
The influence of changes in slenderness ratio on the failure mode.

**Fig 17** illustrates the influence of slenderness ratio on the failure mode of composite columns. As the slenderness ratio increases, buckling becomes more pronounced, and the CFRP–PVC tube tearing location shifts from the lower regions to the mid-span. Furthermore, the peak load decreases with increasing specimen height. Compared with the specimen having a slenderness ratio of 5, the peak load is reduced by approximately 5.1%, 6.2%, and 13.5% for slenderness ratios of 10, 15, and 20, respectively, indicating that higher slenderness ratios have an adverse effect on axial load-bearing capacity.

**Fig 18:**
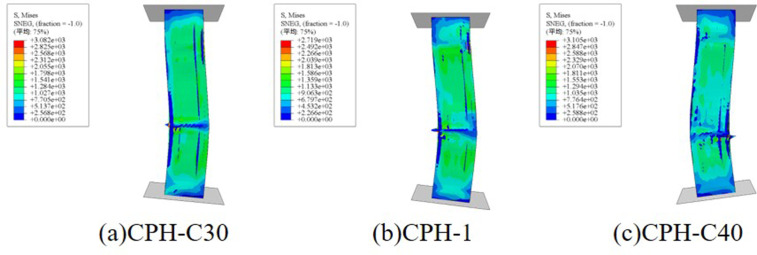
The influence of changes in concrete strength on the failure mode.

**Fig 18** depicts the influence of concrete strength on the failure mode of composite columns. The findings indicate that variations in concrete strength have a negligible effect on failure mode. Compared to C30, the peak load increases by approximately 1.1% for C35, 3.5% for C40, and 18.9% for C60. This suggests that while higher concrete strength contributes to improved load capacity, the enhancement is moderate, indicating that the composite column’s mechanical performance is primarily controlled by the external CFRP-PVC confinement and internal reinforcement.

The slenderness ratio λ of the column specimens was calculated using the expression:


λ=H/B (14)

where *H* is the effective length of the column, *B* is the side length of the cross-section. The calculated slenderness ratios ranged from 5 to 20, indicating that the columns fall into the category of slender members according to GB50010−2010 [22].

## 5. Conclusions

This study introduces the configuration of I-shaped aluminum within CFRP-PVC tube concrete columns to address the issue of poor ductility in concrete-filled CFRP-PVC tube columns, aiming to enhance both performance and durability. The mechanical behavior of these composite columns was investigated through uniaxial compression tests, leading to the following conclusions:

(1) All specimens exhibited typical failure patterns characterized by tearing of the external CFRP-PVC or PVC tubes and flange buckling of the internal I-shaped aluminum sections. As the external confinement increased, the buckling location of the aluminum flange shifted from the mid-span towards the column top, indicating a change in buckling behavior under enhanced restraint.
(2) Specimens confined with a single layer of CFRP showed inadequate confinement, preventing the I-shaped aluminum from yielding and leading to a brittle load-displacement response. At least two CFRP layers are recommended to achieve effective confinement. Moreover, weaker confinement led to a reduced slope in the elastic–plastic phase of the response curve.
(3) The addition of I-shaped aluminum significantly improved both the peak load and ductility of the composite columns by 48.4% and 6.2%, respectively. The synergistic confinement effect provided by CFRP-PVC tubes and the internal aluminum section effectively enhanced the mechanical performance of the system. As the number of CFRP layers increased, the bearing capacity improved by up to 69.8%, while the axial ductility initially declined and then increased, suggesting a non-monotonic influence. Larger CFRP spacing reduced the bearing capacity, though the impact on ductility was minimal.
(4) The introduction of I-shaped aluminum increased the initial axial compression stiffness by 113.1%. Increasing the number of CFRP layers effectively mitigated stiffness degradation and further enhanced initial stiffness. Specimens with different CFRP layer spacings exhibited nearly overlapping axial stiffness curves, indicating similar initial stiffness values.
(5) Increasing the number of CFRP layers and reducing their spacing effectively enhanced the axial compression energy dissipation capacity of the specimens. Notably, the arrangement of CFRP sheets at intervals resulted in a more significant improvement in energy dissipation.
(6) Furthermore, a refined ABAQUS finite element model was developed, and constraint mechanism analysis was conducted based on a validated model. A composite constraint model for CFRP-PVC square tubes and I-shaped aluminum was subsequently proposed.

Despite the promising results, this study has several limitations. First, the experimental program included a limited number of specimens and concrete strength levels, which may restrict the generalizability of the findings. Second, initial geometric imperfections and long-term durability aspects under environmental conditions were not considered. Future work will focus on expanding the parameter range, including higher-strength concretes and varying cross-sectional shapes. Additionally, fatigue performance, long-term durability, and the effects of initial imperfections will be investigated to further validate and optimize the design of CFRP-PVC tube-confined I-shaped aluminum concrete columns.

## Supporting information

S1 File Minimal data set. (RAR)
